# Stiffness of Experimentally Tested Horizontally Loaded Walls and Timber-Framed Modular Building

**DOI:** 10.3390/ma16186229

**Published:** 2023-09-15

**Authors:** Czesław Miedziałowski, Krzysztof Robert Czech, Marta Nazarczuk, Marta Kosior-Kazberuk, Anna Żakowicz

**Affiliations:** 1 Department of Building Structures and Structural Mechanics, Faculty of Civil Engineering and Environmental Sciences, Bialystok University of Technology, Wiejska 45E, 15-351 Bialystok, Poland; c.miedzialowski@pb.edu.pl (C.M.); m.kosior@pb.edu.pl (M.K.-K.); a.zakowicz@pb.edu.pl (A.Ż.); 2 Unihouse SA, Rejonowa 5, 17-100 Bielsk Podlaski, Poland; mnazarczuk@unihouse.pl

**Keywords:** shear walls, spatial modules, modular building, wall stiffness, building stiffness

## Abstract

This paper presents an overview of representative up-to-date research and the authors’ own experimental results from tests of wall elements and a horizontally loaded timber-framed modular building. The research has been conducted in connection with the development of timber-based structures in recent years. In the present research, wall elements and modules of timber-frame construction with life-size dimensions were used. So far, these types of structures have mainly been tested in laboratories—especially with regard to anchoring and cyclic loading. An experimental testing was carried out on a natural scale in two stages based on the standard procedure described in EN 594. In the first stage, wall panels were tested. In the second stage, tests were carried out on a complete four-storey building. Dowel fasteners were used to fix the sheathing to the load-bearing wall structures. Additionally, the sheathing was glued to the timber frame of the walls. The same type of wall element was used for the construction of the tested building. Horizontal loads were applied at the height of the top of the walls in both stages. The building loads were applied in a direction perpendicular to the longitudinal axis of the modules. Based on test data, the stiffnesses of the wall panels and the whole building were derived, as well as the type of interaction between the modules and the influence of the walls on the spatial work of the building. On the basis of the conducted studies, both the stiffness of the walls in different configurations and the stiffness of the complete building were determined, as well as the nature of the interaction of neighbouring modules and the influence of wall connections on the 3D working of the building. The results show that the stiffness of the building in the horizontal plane in the direction of the applied load is almost twice as high as the sum of the stiffnesses of the building walls in the same direction.

## 1. Introduction

**Figure 1:**
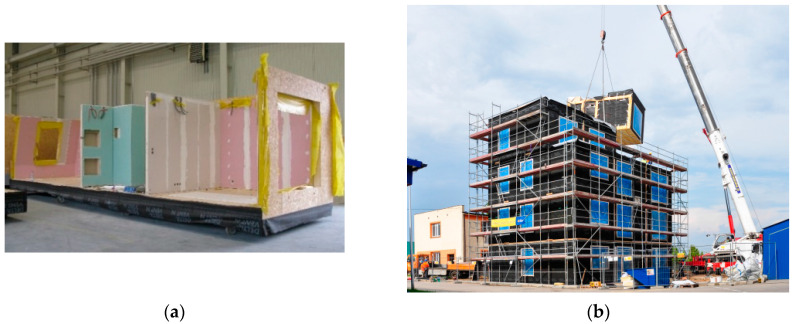
Example of realisation of a multi-storey building from modular elements: (**a**) prefabrication of modules in a factory and (**b**) assembly of a building from modular elements on a construction site.

Timber-based construction is considered to be very ecological and beneficial in the current period of socio-economic development. Among the many types of timber-based construction, one of the latest trends are modular buildings. Their history dates back to the 1950s. The prototype of this type of construction was mobile transport containers. Today, timber modular buildings are made as spatial large-scale structures consisting of floor, walls and ceiling panels [1,2,3]. Mostly, such elements are manufactured in the factory, with external and internal surface finishes, fitted carpentry, complete sanitary and electrical installations, and even utility fittings for kitchen and bathroom modules. Modules can be of timber frame or plate structure, i.e., CLT (cross-laminated timber) [1,4,5]. The dimensions of modular elements depend on the size of the building and the transport gauge of transport routes. An example of a spatial module during its prefabrication in the factory and the assembly of modules on the construction site is shown in Figure 1.

**Figure 2:**
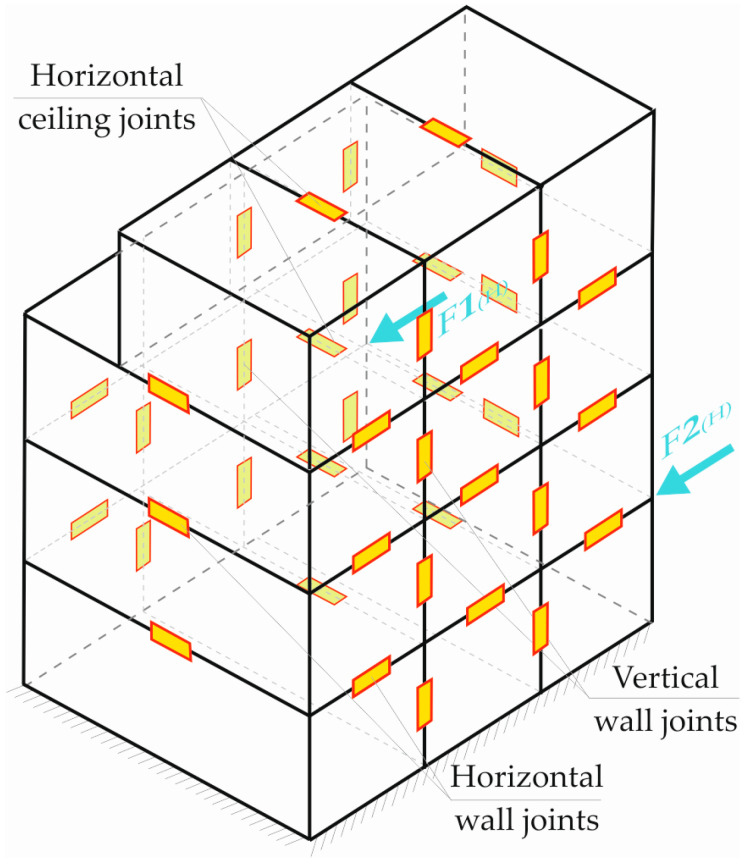
Types of inter-module connections in the building and the considered direction of interaction of the elements.

One of the requirements for the exploitation of this type of building is its stiffness, especially for horizontal loads, which largely depend on the inter-module connections [6,7]. An example of the location of such inter-module connections is shown in Figure 2. 

Buildings and their individual components are spatial structures and work in 3D static schemes. The computational and design models should also consider the 3D working of the structure. The difficulty is that these are complex systems—that is, structures made up of many different elements (columns, beams, sheathing, connectors in the elements and connections between elements). Computational models should therefore be compulsorily validated by experimental studies.

There are relatively few published experimental studies on the spatial work of buildings. Considerably more research has been caried on wall elements.

Comprehensive experimental research on walls was conducted by Kamiya F. [8], Stewart A.H. et al. [9], Sugyiama H. et al. [10] and Baszeń M. et al. [11], among others. Recently, numerical analyses as well as experimental studies of walls with openings were carried out by Kuai L. [12].

In the studies of Martin Z.A. et al. [13], Šilih E.K. and Premrov M. [14], more attention has been paid to the study of wall elements with openings.

Parallel to experimental research on walls, studies of timber frame and sheathing connectors were conducted. In 1949, in the USA, regulations were introduced to allow the use of sheathing other than timber cladding [15].

A broad review of the literature related to the testing of walls both with and without openings was caried out in a paper by Miedzialowski Cz. and Malesza M. [16]. At present, the guidelines of the EN 594 standard of July 2011 [17] apply to testing of stiffness and load-bearing capacity of wall panels.

The first comprehensive testing of buildings started with structures erected traditionally, i.e., from linear elements. This type of research was carried out by Tuomi R.L. and McCutheon J. [18], Boughton G.N. and Reardon G.F. [19] and Boughton G.N. et al. [20], among others. 

The subject of the studies by Collins et al. [21] and Malesza et al. [22] were single-family prefabricated panel buildings.

Prefabricated timber-frame modules were the object of research and numerical analyses by Smith J. et al. [23]. Experimental and numerical studies of timber-frame modules have also been carried out by Ormarsson S. et al. [24,25]. The horizontal arrangement of two modules was studied by Montaño et al. [26]. The advantages and limitations associated with modular construction of multi-family buildings are described in the work of Cameron P.J. JR and Di Carlo N.G. [1].

Popovski M. et al. [27] conducted a study of a two-storey CLT modular building. Three-storey and seven-storey buildings, also made of CLT, were the subject of laboratory tests (mainly under cyclic loading conditions) carried out by Ceccotti A. et al. [28,29]. Building tests were also analysed in the work of Li et al. [7]. 

An extensive review of studies of various modular building structures was performed by Lacey et al. [30]. 

Most recently, Cowled C. J. L. et al. [31] tested timber-frame shear walls with additional gypsum boards under monotonic loading, similarly to Valdivieso et al. [32] where they also performed cyclic loading tests. Kuai L. et al. [33] developed elasto-plastic model of timber-frame shear walls with openings and validated it by testing it under cyclic loading. Abrahamsen R. et al. [34] participated in Dyna TTB project that focused on an identification of mechanical properties of tall timber buildings by performing dynamic tests on existing objects. Amaddeo C. and Dorn M. [35] tested a timber-frame building against vibrations. As can be seen from the literature review, there is a lack of research results for modular timber-frame buildings. The paper will present both the tests of timber-frame walls, with I-joists timber studs and OSB sheathing (connected to the studs with staples and additionally glued to the timber-frame structure), and tests of a complete modular building made of the above-described frame walls. As a result of the conducted analyses, the stiffness of the walls and the building will be determined under the conditions of horizontal force applied at the top of the walls in the direction perpendicular to the length of the modules. 

The study will also analyse the effect of openings in the walls and the location of particular wall types on the final stiffness of the building. The influence of module connections on the spatial behaviour of the building will also be analysed. The conclusions will indicate the possibilities of practical use of the results of the conducted research.

The aim of this paper is therefore to supplement the study of timber-frame modular buildings and to obtain experimental data to validate the numerical models. The research hypothesises that the stiffness of the building is significantly influenced by the connections between modules, the arrangement of load-bearing walls and the area and arrangement of window and door openings in the modules.

Such an experimental study of natural-scale structures will be innovative considering the current state of research in this topic and its relevance for the correct assessment of the behaviour of timber-frame modular houses. It will be a substantive contribution to the static working theory of timber-frame modular buildings.

## 2. Construction of Tested Walls and Building

The building’s structure was constructed using timber-frame technology. It is a four-storey building realised by Unihouse SA [36], a leading manufacturer of wooden buildings in Poland. Every storey comprises three modules. One of the modules is designed as a one-bedroom studio. Another two modules are combined to form a two-bedroom flat. 

On the top floor there is one large three-room unit with a large terrace on the south side. Each unit is accessed from an external gangway. The roof is flat and made of prefabricated panels.

**Figure 3:**
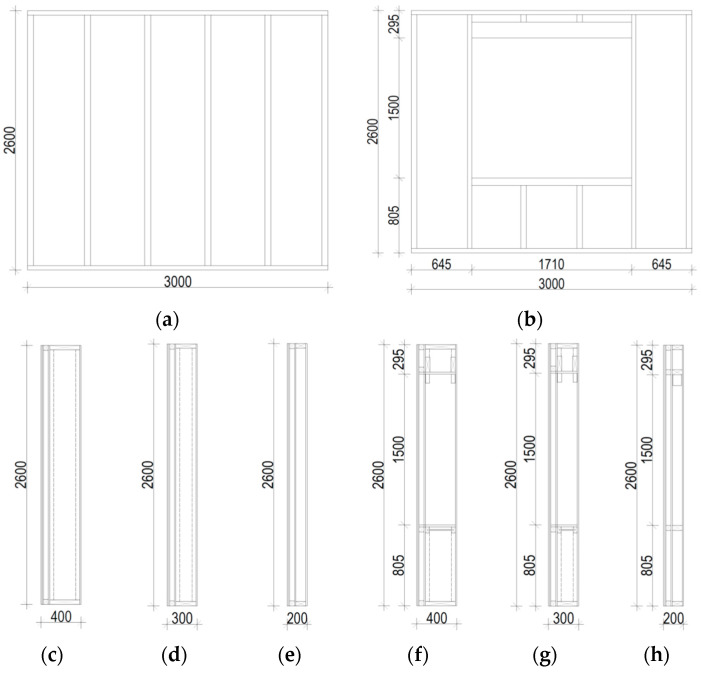
Tested wall panels [38]: (**a**) panel without openings, (**b**) panel with an opening, (**c**) section of the SZA wall without openings, (**d**) section of the SZB wall without openings, (**e**) section of the SZD wall without openings, (**f**) section of the SZA wall with an opening, (**g**) section of the SZB wall with an opening and (**h**) section of the SZD wall with an opening.

**Figure 4:**
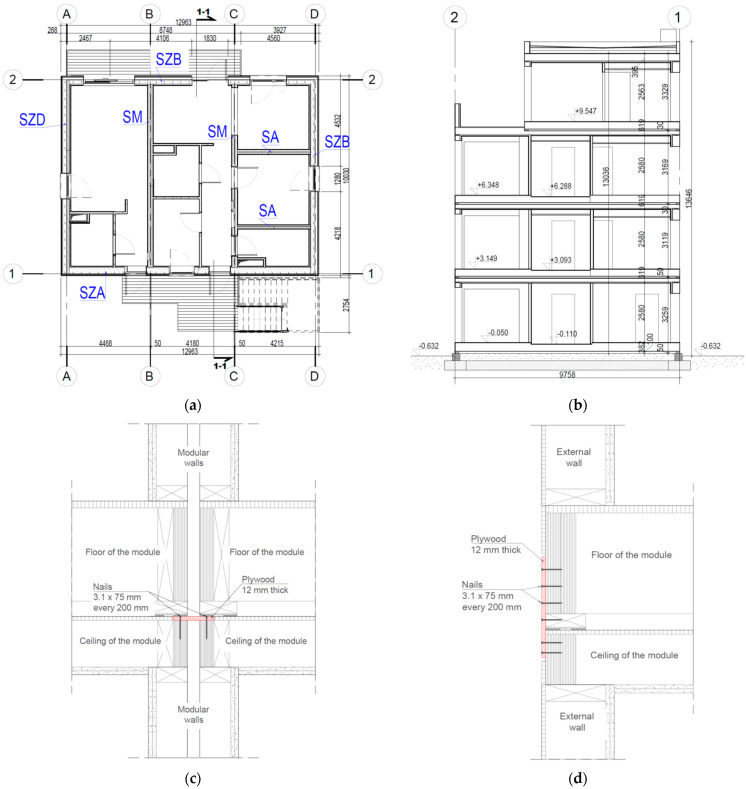
Plan and cross section of the tested building and module connection diagrams [39]: (**a**) ground floor plan, (**b**) vertical section, (**c**) horizontal connections between modules and (**d**) vertical connections between modules.

The single module is designed as a three-dimensional element. Its stiffness is provided by two horizontal plates (sheathed ceiling and sheathed floor), longitudinal and transverse external walls and internal walls, if possible. At the level of the ceiling and at the floor level, around the perimeter of the module, continuous timber beams were used (connected only at their corners). The individual modules are constructed from five wall types: type A external wall (marked as SZA), type B external wall (SZB), type D external wall (SZD), type M inter-module walls (SM) and type A internal walls (SA)—according to Figure 3 and Figure 4a. Depending on the type of wall, the load-bearing structure consists of timber I-joist studs with a section hight of 300 mm or 200 mm, or 50 × 100 mm C24 or LVL timber studs different for different floors [37]. The top and bottom rails of the walls are made of LVL timber. Structural sheathing of walls is 12 mm OSB 3 boards or 12.5 mm structural gypsum boards. Internal load-bearing walls (type SA) are made of 100 mm LVL or C24 timber studs and LVL timber top and bottom rails. The sheathing of walls is made of 12.5 mm structural gypsum boards.

The structure of ceilings comprises timber joists sheathed with OSB3 boards. On the perimeter of the panel, there are continuous 150 mm LVL timber beams (connected only in corners).

The floor structure of the intermediate storey in the bathrooms is made up of 240 mm timber I-joists. In the other rooms, the heights of the joists are 300 mm. On the perimeter of panels there are LVL timber beams. Structural joists are sheathed with OSB3 boards [37].

Lintels in walls are made of timber beams.

The flat roof is ventilated, sloping in one direction. Its structure is 300 mm timber I-joists sheathed with OSB3 boards. On the perimeter of the panel there are continuous 300 mm timber beams (as before, connected only in corners).

During tests, there were no doors or windows in the building. The external staircase was designed as a steel structure.

The above-mentioned three types of walls (with or without openings) are presented in Figure 3.

The panels, depending on the type of wall, differed in the type of fasteners fixing the sheathing to the structural beams (framing):

- type 1: 1.53 × 50 mm staples, with 70 mm spacing;
- type 2: 1.53 × 50 mm staples, with 120 mm spacing;
- type 3: 3.5 × 35 mm screws, with 70 mm spacing.

Additionally, the sheathing in all walls was glued to the framing. The use of glue was intended to improve the airtightness of the building.

The form of the walls was also diversified—three panels in each group were solid, while one had an opening of 1710 × 1500 mm.

The ground floor plan and vertical section of the building under study are shown in Figure 4a,b, respectively. The connection diagrams of the modules are shown in Figure 4c,d. Between modules, board type connections of 12 mm plywood were used.

## 3. Experimental Research Procedures

The wall and building test methodology was adapted by analogy with the wall panel test guidelines according to EN 594 “Timber structures. Test methods. Racking strength and stiffness of timber-frame wall panels” [17].

The loading rate should ensure that 90% of the wall load-bearing capacity is achieved within 300 ± 120 s.

**Figure 5:**
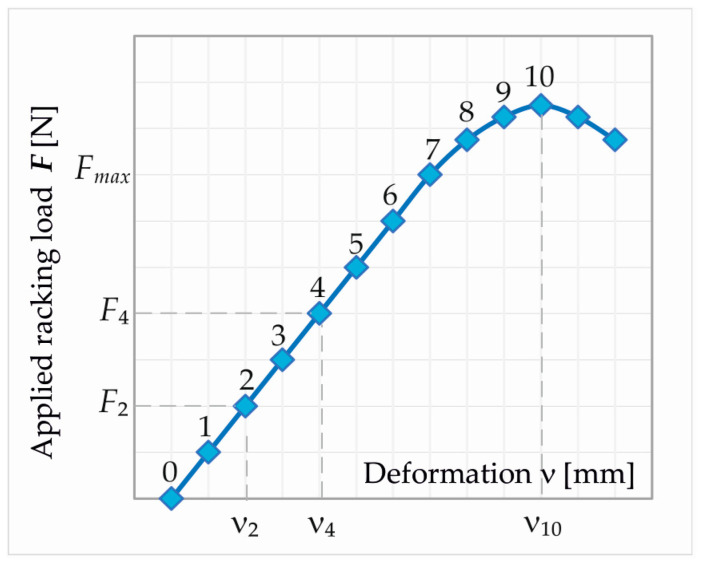
Wall loading process according to EN 594 [17].

Wall panels’ loading process ends with the collapse of the walls, according to the diagram presented in Figure 5.

According to the standard [17], the stiffness of the wall is calculated based on the formula:

(1) $$R=\frac{F_{4}-F_{2}}{\nu_{4}-\nu_{2}},\;\left[N/mm\right]$$

where:

*F*_2_—racking load of 0.2 *F_max_*;

*F*_4_—racking load of 0.4 *F_max_*;

*v*_2_, *v*_4_—deformations corresponding to *F*_2_ and *F*_4_ forces.

## 4. Experimental Test Rig and Measuring Equipment

### 4.1. Wall Panels Tests

**Figure 6:**
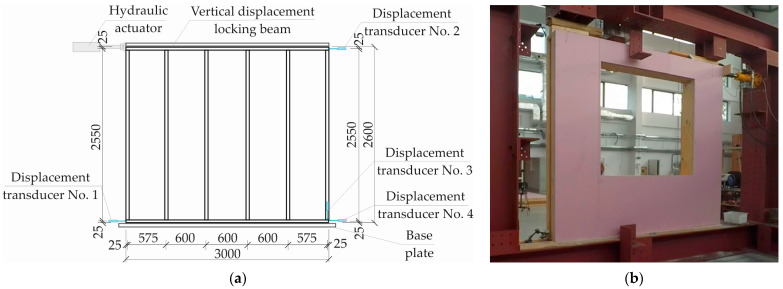
Experimental test rig for wall panels: (**a**) test rig scheme and (**b**) view of the test rig.

Destructive testing of wall panels was conducted in the laboratory hall of the Faculty of Civil Engineering and Environmental Sciences of Bialystok University of Technology [38]. Wall panels were fixed to the experimental test rig shown schematically in Figure 6a. A wall panel with an opening during testing is shown in Figure 6b.

During tests, wall panels were loaded from the top with a head binder beam blocking vertical displacements. From the bottom, panels were fixed to the test rig with screws spaced at 60 cm (Figure 6a). 

The load was applied in a horizontal direction with a multi-channel hydraulic system dedicated to structural loads application (HYSDOZOK). The tests used one of the available hydraulic cylinders (with a loading range of ±200 kN). The force increment during the test was 5 kN every 30 s. The loading process was continued until the wall failed (*F_max_*) [38]. 

During the load application, the force applied to the wall panel and its displacement at selected measurement points were registered. For this purpose, a 16-channel computerised vibration analyser KSD-400 (P.U.P. SENSOR, Łódź, Poland) was used with a 12-bit AI converter card of the DAQ Card-700 type from National Instruments (Austin, TX, USA). The force and displacements were recorded at a rate of 16 samples per second.

**Figure 7:**
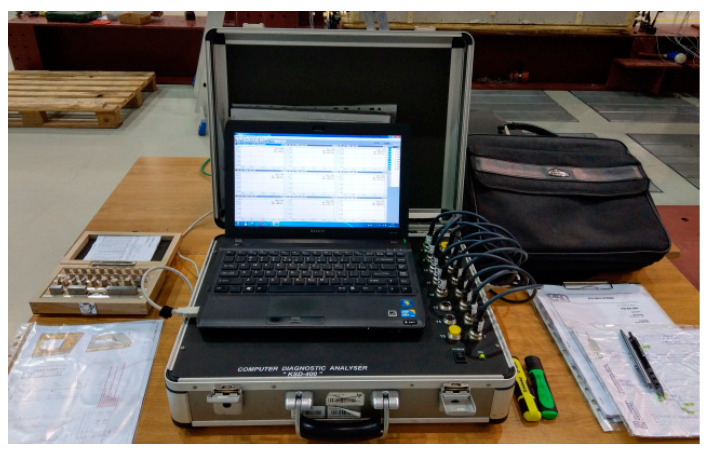
View of the data acquisition and recording station.

The measurement data acquisition and recording stand is shown in Figure 7.

Displacement measurements of the wall panels were carried out using three potentiometric transducers type SPR18-50 (MEGATRON Elektronik GmbH & Co. KG, Putzbrunn/Munich, Germany) with a 50 mm measuring base and a linearity tolerance of ±0.1% and one transducer type SPR18-75 (MEGATRON Elektronik GmbH & Co. KG, Putzbrunn/Munich, Germany) with a measuring base of 75 mm and identical linearity. The accuracy of the readings of the displacement transducers connected to the KSD-400 measurement system was verified before the measurements were carried out. A set of calibration steel plates (no. 180 492) was used for this purpose. The maximum measurement error did not exceed 3.8%. The force signal was read directly from the structural load application system (HYSDOZOK) and also recorded using the KSD-400 recorder.

As can be seen in Figure 6a, transducers No. 1, 2 and 4 were mounted to measure horizontal displacements in the plane of the wall. The task of displacement transducer No. 3 was to measure vertical displacement in the plane of the wall, taking into account the compression of the wall to the base plate.

### 4.2. Building Tests

When planning the experimental testing of the building, it was assumed that in order to meet the stated objective and the requirements of the company enabling the testing (Unihouse SA), the building could not be significantly damaged. The tests had to be conducted in such a way as not to cause permanent damage to the building structure. At the same time, it was desirable to obtain significant displacements of the building. This could be accompanied by minor damage to the building finishes (for example, plastering cracks) and audible crackling in the structure. Based on preliminary analyses and previous wall tests, the permissible displacement of the building in the horizontal plane was determined to be about 25 mm (ca. 1/500th of the building hight *H*) and the load-bearing capacity to be about 500 kN of force applied at the level of the ceiling above the ground floor [39].

**Figure 8:**
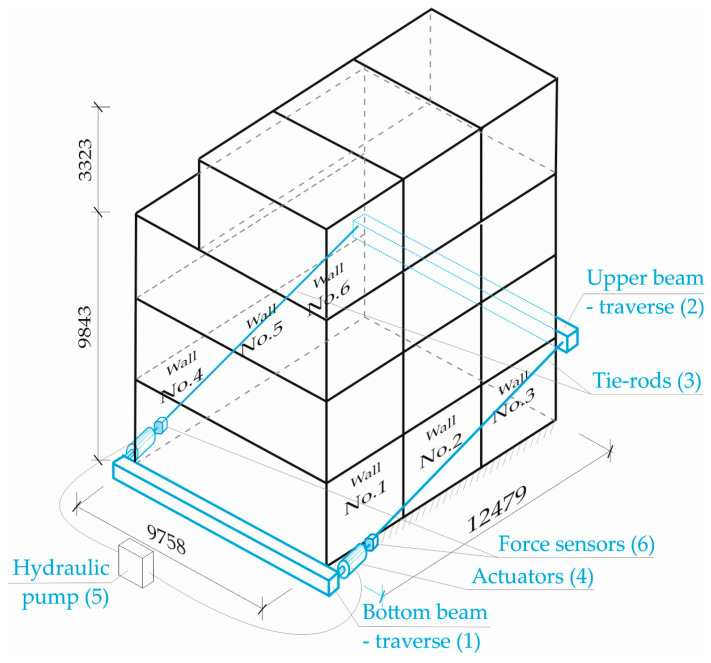
Schematic of the test rig for building loading.

Figure 8 schematically shows the way in which the loads were planned.

The figure above shows external walls No. 1 to No. 3 (type SZA) and No. 4 to No. 6 (type SZB), which mainly took up the loads transmitted from the hydraulic actuators. The interior walls (type SA), which also took up part of the loads, are not visible in Figure 8. Their location in the building can be seen in Figure 4a.

**Figure 9:**
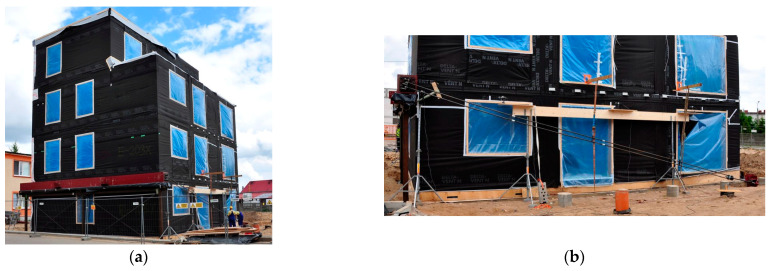
View of the tested building and the load application system [Fot. Unihouse SA]: (**a**) upper beam, traverse and (**b**) tie rods.

**Figure 10:**
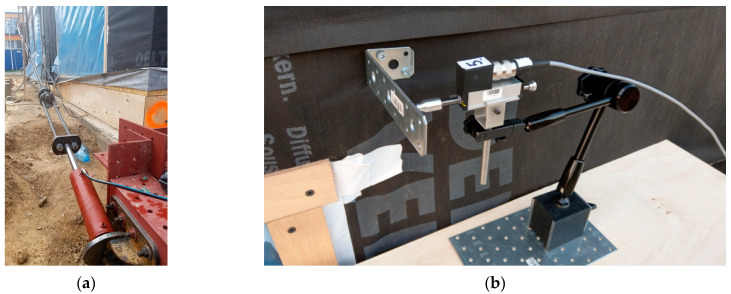
Force application and displacement registration systems: (**a**) actuator and force sensor and (**b**) displacement transducer.

The building under test and the technical instrumentation used during the measurements are shown in Figure 9 and Figure 10.

The load application system used was meant to apply horizontal loads on the building from the gable wall side and to implement assumed loading in stages (Figure 8). Loads were applied through traverse beams (1–2) placed at the level of the building foundation and at the level of ceiling above the ground floor, respectively. Traverses (1–2) were connected with tie rods (3). Loads were applied with hydraulic actuators (4) placed symmetrically on tie rods on both sides of the building. Hydraulic actuators were powered by a hydraulic pump (5).

The force values in tie rods were registered with force transducers (6), which were also mounted on the tie rods. Walls displacements were registered with displacements transducers.

The force and displacement measurements used a 16-channel KSD-400 computerised vibration analyser from P.U.P. Sensor (the same one used in the wall panel tests), two CL18-type force transducers (from ZEPWN, Marki, Poland) and a set of eight potentiometric linear displacement transducers from Megatron:

- type SPR18-25 (MEGATRON Elektronik GmbH & Co. KG, Putzbrunn/Munich, Germany) with a measurement range of 25 mm and linearity tolerance of ±0.2% (4 pieces);
- type SPR18-50 (MEGATRON Elektronik GmbH & Co. KG, Putzbrunn/Munich, Germany) with a measurement range of 50 mm and linearity tolerance of ±0.1% (4 pieces).

The measurement error for the displacement transducers wired to the KSD-400 signal recorder, was determined during tests using a set of calibration steel plates (no. 180 492). The maximum indication error of the complete measuring system did not exceed 4.3%.

**Figure 11:**
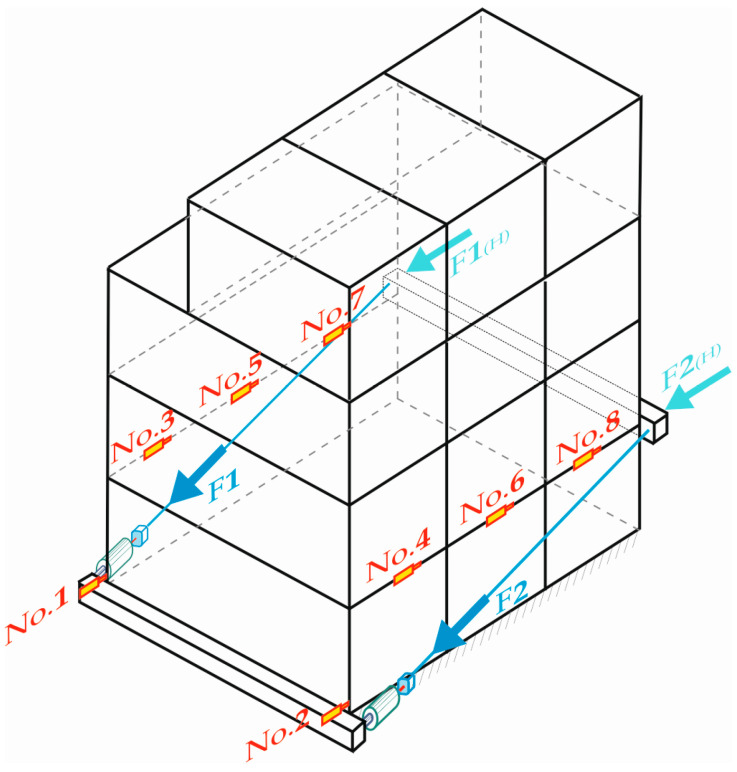
Schematic location plan of potentiometric linear displacement transducers and hydraulic actuators.

Displacement transducers marked as No. 1 and No. 2 were placed directly above the foundation of the building, from the north-eastern side. Transducers No. 3 to No. 8 were placed around 30 cm above window lintels. The location of potentiometric linear transducers with its numeration (marked from No. 1 to No. 8) is shown schematically in Figure 11. In the figure below, the location of hydraulic actuators is marked as F1 and F2.

The tests were organized in such a way that enabled full coordination of all activities during measurements, such as implementation of load stages, forces and displacements values registration and technical state of the building supervision (displacement values, crackles, cracks). 

**Figure 12:**
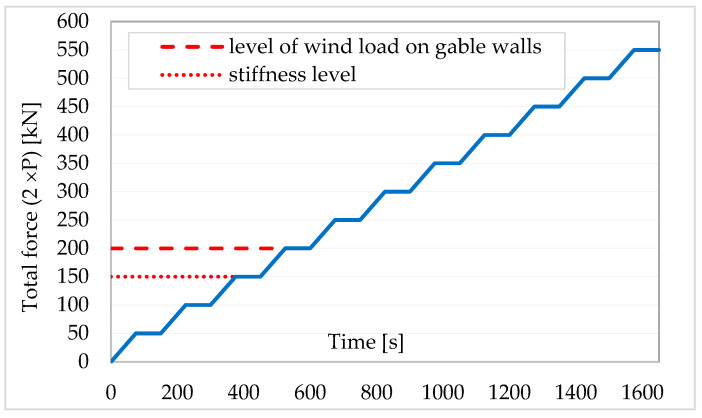
Assumed load implementation plan [39].

Loads were implemented according to a static scheme shown in Figure 12. 

## 5. Test Results

### 5.1. Test Results for Wall Panels

**Figure 13:**
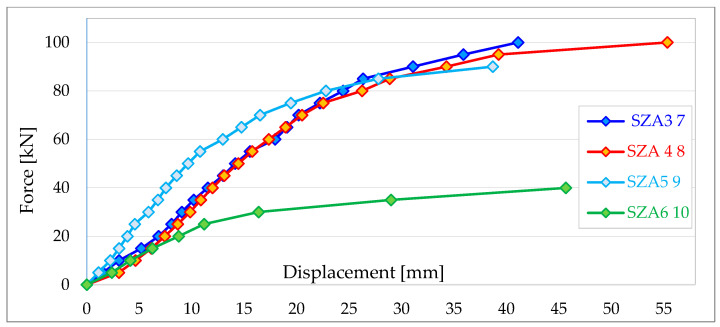
Force–displacement diagram for SZA-type external walls [38].

Figure 13 shows an example of force–displacement diagrams obtained during testing of Type A external wall panels (designation SZA). Only the horizontal displacements in the plane of the panel recorded using displacement transducer No. 2 are summarised in the figure.

The research described in detail in [38], shows that walls with openings have only about 30–40% of the load-bearing capacity of walls without openings. These values are about 40–50 kN for each group of walls. However, the load capacity of walls without openings within all groups is similar (regardless of the type of fasteners) and does not exceed 145 kN.

External walls with structural gypsum boards sheathing (SZA type) have a lower load-bearing capacity than walls with OSB3 boards sheathing, which is quite obvious since wood-based boards have higher stiffness than gypsum boards.

The stiffness of the walls, according to the test results summarised in paper [38], ranges from 3791 N/mm to 6186 N/mm in the case of SZA-type walls, from 2316 N/mm to 4912 N/mm in the case of SZB-type walls and from 1715 N/mm to 5652 N/mm in the case of SZD-type walls.

Detailed results of stiffness calculations according to EN 594 standard [17] for selected walls used in the analysis of tested building are presented in Table 1.

The test results confirm that the stiffness of walls significantly depends on their configuration. Panels with openings are the least rigid in each of the considered groups.

In timber-frame prefabricated modular buildings in the longitudinal direction of the building and transverse to the modules all walls usually have large door and window openings, affecting the stiffness and operation of the structure; this influences the stiffness and structural behaviour of the building, as shown in Figure 4a.

### 5.2. Building Test Results

**Figure 14:**
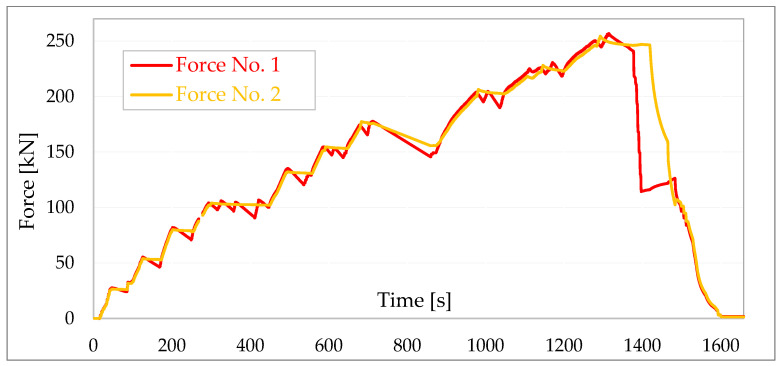
Diagram of forces in tie rods [39].

**Figure 15:**
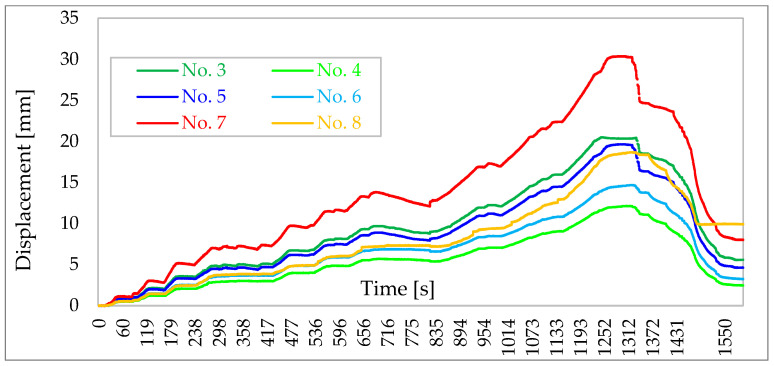
Diagram of wall displacements [39].

A diagram of the forces in the tie rods during the building tests is shown in Figure 14 and a diagram of the displacements of individual walls is shown in Figure 15.

**Figure 16:**
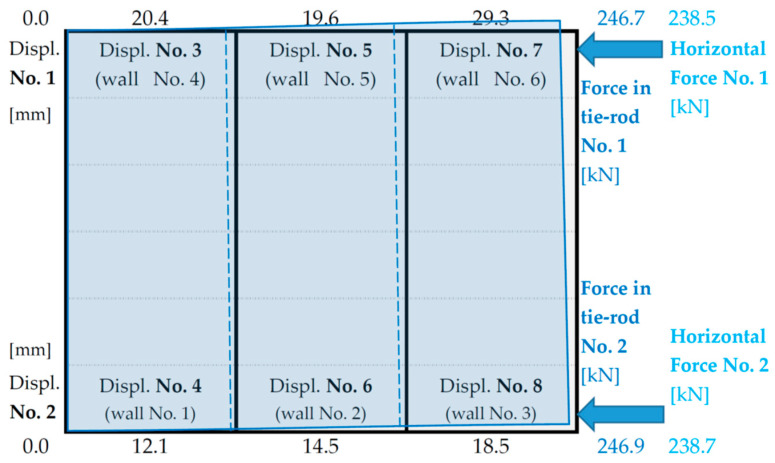
A schematic drawing of the displacements of the building walls corresponding to the maximum forces in the tie rods.

A detailed summary of the displacement values for each measurement point as a function of the applied loads is given in Table 2 and Table 3 and schematically shown in Figure 16.

**Figure 17:**
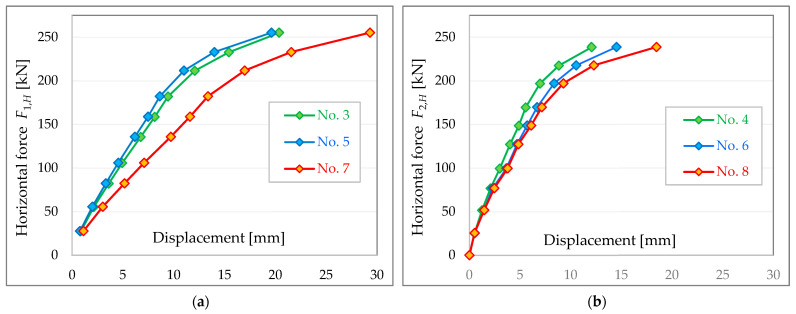
Displacements of the building walls in a longitudinal direction: (**a**) along the line of force *F*_1*H*_ and (**b**) along the line of force *F*_2*H*_.

Displacement curves for individual measurement points as a function of applied loads in the horizontal plane (projected forces in tie rods) are shown in Figure 17.

On the basis of the results of the displacement tests, the sum of the average stiffness of the walls on the left and right sides of the building and the average total stiffness of all the walls were determined, according to the procedure specified in EN-594 [17]. The obtained results are summarised in Table 4.

## 6. Discussion

The tests proved that, with increasing loads, there is a continuous increase in the displacement of all elements, in accordance with their structure and geometry.

The results presented in Table 4 show a significantly lower summed stiffness of the walls of the left side of the building with a significantly larger area of openings (*R_W,L_* = 15,048 N/mm) compared to the stiffness of the walls of the right side (*R_W,R_* = 22,810 N/mm). In the analysed case, the difference is as high as 34.0%.

The recorded building displacements confirm the significantly lower stiffness of the left side (measuring points No. 3, No. 5 and No. 7) than the right side (measuring points No. 4, No. 6 and No. 8)—as indicated by the torsion of the structure visible in Figure 16.

Deformations of timber-framed buildings, unlike traditional structures, are also highly dependent on the internal inter-elemental vertical and horizontal connections.

As conducted tests show, the horizontal displacements of individual wall panels at failure do not exceed 56 mm.

The displacements values of the tested building are not that high and range from a few to several tens of millimetres (maximum 29.3 mm). For the wind load level (according to Figure 12 approximately 75 kN), the displacement values of the walls, on the left and right sides of the building, are between 2.5 to 5.2 mm (Figure 16). At the so-called stiffness level (according to Figure 12 about 100 kN), the displacements values are around 3.8–7.1 mm (Figure 16).

The total stiffness of the walls in the building was assumed according to the tests without considering their interaction. For untested walls, the stiffness was interpolated in proportion to the stiffness of full wall segments (based on the stiffness of tested walls of the same type).

*R_W_ = R_SZB_*_(*A-B*)_ + 2 · *R_SZB_*_(*B-C*)_ + 2 · *R_SA_* + *R_SZA_*_(*A-B*)_ + *R_SA_*_(*B-C*)_ + *R_SZA_*_(*C-D*)_
 = 986 + 2 · 1577 + 2 · 2913 + 3198 + 1866 + 4264 = 19,294 [N/mm].

The average stiffness of the whole building according to the data in Table 2 is *R_B_* = 36 530 N/mm.

The first crackles in the structure were registered at loads 2·P = 100 kN load. The first drywall cracks occurred at 2·P = 150 ÷ 200 kN load.

## 7. Conclusions

Wall and building stiffness tests were carried out according to the methodology outlined in EN 594 standard [17]. Tests on walls were continued up to their failure, while tests on the building were carried out up to the stage of non-linear behaviour, cracking and first cracks in the finish (plaster). 

As a result of the tests and comparative analyses, it should be concluded that:

- the stiffness of the walls is significantly dependent on their configuration and the surfaces of the openings, and ranges from 1715 N/mm to 6186 N/mm;
- the walls of the left side of the building with a significantly larger area of openings have a total stiffness 34.0% lower than the total stiffness of the walls of the right side (the reason for the torsion of the building visible in Figure 16);
- the total stiffness of the building walls in the direction of the applied loads (*R_W_* = 19,294 N/mm) is 17,200 N/mm less than the spatial stiffness of the building (*R_B_* = 36,530 N/mm)—showing how important the influence of the connections between the modules is on the overall stiffness of the building;
- the configuration and location of the walls in the modules influences the nature of the work and deformation of the building and, consequently, the distribution of horizontal loads to the individual walls.

The next stage of the research will be the development of numerical models of three-dimensional modules and entire buildings.

The results obtained are novel in terms of natural-scale testing of elements and buildings of frame and modular construction. In addition to their scientific value, the results will serve to validate three-dimensional numerical computational models—as only validated models can be used to determine reliable deformations and real internal forces in particular structural elements of buildings.

## Figures and Tables

**Table 1:** Stiffness and horizontal load-bearing capacity of the tested panels [38].

No. of Wall Panel	*F_max_* [kN]	*F*_2_ [kN]	*F*_4_ [kN]	*ν*_2_ [mm]	*ν*_4_ [mm]	*R* [N/mm]
SZA3 7	100	20	40	6.85	11.54	4264
SZA6 10	40	8	16	4.15	6.26	3791
SZB6 14	55	11	22	7.60	12.35	2316
SZD4 16	120	24	48	10.52	18.76	2913
SZD5 17	145	29	58	8.99	16.67	3776

**Table 2:** Summary of the forces in the tie-rod No. 1 and the corresponding displacements of the left side of the building.

Force in Tie Rod *F*_1_ [kN]	Horizontal Force *F*_1,*H*_ [kN]	Horizontal Displacement [mm]		
		No. 3	No. 5	No. 7
0	0.0	0	0	0
26.7	25.8	0.85	0.78	1.12
53.7	51.9	2.12	1.99	3.02
79.6	76.9	3.59	3.32	5.16
102.4	99.0	4.91	4.54	7.09
131.3	126.9	6.73	6.18	9.72
153.5	148.4	8.11	7.47	11.60
176.1	170.2	9.42	8.63	13.37
204.6	197.8	12.05	11.01	16.98
225.1	217.6	15.41	13.99	21.56
246.7	238.5	20.36	19.62	29.30

**Table 3:** Summary of the forces in the tie rod No. 2 and the corresponding displacements of the right side of the building.

Force in Tie Rod	Horizontal Force	Horizontal Displacement [mm]		
*F*_2_ [kN]	*F*_2,*H*_ [kN]	No. 4	No. 6	No. 8
0	0.0	0	0	0
26.3	25.4	0.49	0.53	0.51
53.5	51.7	1.23	1.46	1.46
79.5	76.8	2.07	2.30	2.46
103	99.6	2.98	3.65	3.77
131.6	127.2	3.99	4.60	4.84
153.8	148.7	4.86	5.70	6.10
175.5	169.6	5.54	6.68	7.15
203.8	197.0	6.95	8.36	9.28
225.1	217.6	8.81	10.54	12.28
246.9	238.7	12.05	14.51	18.46

**Table 4:** Stiffness of walls and building.

Part of the Building	*F_max_* [kN]	*F*_2_ [kN]	*F*_4_ [kN]	*v*_2_ [mm]	*v*_4_ [mm]	*R* [N/mm]
Left side walls	238.5	51.9	99.0	2.37	5.50	15,048
Right side walls	238.7	51.7	99.6	1.40	3.50	22,810
All walls/entire building	477.2	103.6	198.6	1.88	4.50	36,530

## Data Availability

Data available on request due to privacy.
